# Seaweed Sulfated Polysaccharides against Respiratory Viral Infections

**DOI:** 10.3390/pharmaceutics13050733

**Published:** 2021-05-16

**Authors:** Mehwish Jabeen, Mélody Dutot, Roxane Fagon, Bernard Verrier, Claire Monge

**Affiliations:** 1 Laboratory of Tissue Biology and Therapeutic Engineering, UMR5305 Centre National de la Recherche Scientifique/Université Claude Bernard Lyon 1, 7 Passage du Vercors, CEDEX 07, 69367 Lyon, France; mehwish.jabeen@ibcp.fr; 2 Recherche & Développement, Yslab, 29000 Quimper, France; melody.dutot@yslab.fr (M.D.); roxane.fagon@yslab.fr (R.F.)

**Keywords:** seaweed, sulfated polysaccharide, respiratory virus, approved antivirals, antiviral activity, mechanism of action

## Abstract

Respiratory viral infections have been a leading cause of morbidity and mortality worldwide. Despite massive advancements in the virology field, no specific treatment exists for most respiratory viral infections. Approved therapies against respiratory viruses rely almost exclusively on synthetic drugs that have potential side effects, restricting their use. This review aims to present natural marine sulfated polysaccharides possessing promising antiviral activity against respiratory viruses that could be a safe alternative to synthetic broad-spectrum antiviral drugs. The antiviral properties of marine sulfated polysaccharides are presented according to their mechanism of action on different types and strains of respiratory viruses, and the potential limits of their use are discussed.

## 1. Introduction

The respiratory tract is one of the main portals of entry for human viruses. Respiratory tract infections lead to enormous health and economic burdens and cause severe outcomes, including hospitalization and death, especially in developing countries [1,2,3,4]. In 2013, the WHO’s Battle against Respiratory Viruses initiative (BRaVe) recognized acute respiratory infection as a major global public health issue [5]. Unfortunately, nearly ten years later, respiratory infections are still a global health security threat, exemplified by the recent coronavirus outbreak in 2020 [3].

Viral respiratory tract infections (vRTIs) can be due to several families of viruses, such as picornaviruses (human rhinoviruses (HRV)), coronaviruses (CoV), ortho- and paramyxoviruses (influenza virus, parainfluenza virus (PIV or HPIV for human), human metapneumovirus (HMPV) and respiratory syncytial virus (RSV)), adenoviruses and herpes viruses [1,6]. Since the beginning of the 21st century, the world has faced several episodes of epidemic or pandemic respiratory illnesses due to the emergence of new respiratory viruses, including severe acute respiratory syndrome coronavirus (SARS-CoV) in 2003, H1N1 influenza in 2009, avian influenza A viruses, such as H5N1 and H7N9, middle east respiratory syndrome coronavirus (MERS-CoV) in 2012, and the recent SARS-CoV-2 in 2019. These reemerging and highly pathogenic pandemic viruses have been one of the leading causes of morbidity and mortality worldwide and are known to cause acute respiratory infections in all age groups globally and all year round [7,8,9,10]. In 2017, it was estimated that more than 54 million lower respiratory tract infection (LRI) episodes were due to influenza worldwide, leading to more than 9 million hospitalizations and 145,000 deaths among all ages [11]. Even though all age groups are susceptible to influenza, children up to 5 years, pregnant women, the elderly and individuals with specific chronic medical conditions are considered at-risk populations. Alike influenza-virus-associated infections, RSV caused around 1.5 million episodes of acute LRIs in 2015 in the elderly, specifically those aged 50–64 years [12]. Although other respiratory viruses, such as adenovirus and rhinovirus, cause lower mortality, they are associated with significant morbidity, which causes a huge economic burden. Increased respiratory viral infections worldwide necessitate searching for safe and effective antiviral drugs to counteract these massive healthcare problems.

## 2. Symptoms of Respiratory Viral Infections and Approved Therapies

**Figure 1:**
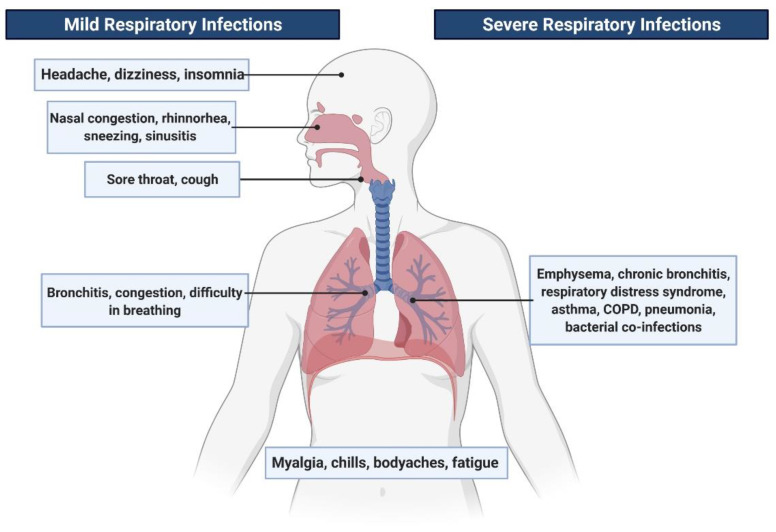
Symptoms related to viral respiratory tract infections.

The transmission of viral pathogens occurs mainly by the respiratory route, characterized by aerosol emission or droplets primarily during a cough or sneezing episode [6,13]. In most cases, these vRTIs are limited to the upper respiratory tract but can lead to acute lower respiratory tract infections as well, particularly in the case of RSV, influenza virus, PIV and SARS-CoV2 [14,15,16]. The symptoms of viral respiratory infections can include congestion of either the nasal sinuses or lungs, runny nose, cough, sore throat, body aches, fatigue (Figure 1).

In the most severe cases, symptoms can be chills, difficulty in breathing, dizziness, bronchiolitis, asthma exacerbations, pneumonia, respiratory distress syndromes and chronic obstructive pulmonary disease (COPD) [5,17]. The propagation of several respiratory viruses has also been reported from the respiratory tract to the central nervous system (CNS) [18].

Although some viruses may be associated with defined clinical signs and symptoms, there is also some intersection in the correspondence of pathology and the responsible pathogen. It is thus difficult to define viral respiratory diseases because the symptoms related to vRTI commonly overlap [6]. For example, the common cold may originate from different etiologic agents, such as rhinoviruses or coronaviruses. Similarly, influenza may be caused by the influenza virus, but also by RSV, *Haemophilus influenzae* type b, or *Streptococcus pneumoniae* [11].

Symptomatic therapies or antiviral medications are still the major tools to treat vRTIs [19] as vaccines are currently not yet available for most of the respiratory viruses [20] except against influenza, adenovirus and more recently, against SARS-CoV-2. However, it is also important to notice that such vaccines will face several specific hurdles: (i) they could be restricted to some countries and dedicated personnel, as evidenced by the adenovirus vaccine available only to military personnel in the US; (ii) their mild efficacy (from 30% to 60% for the influenza vaccine) and need for annual revaccination due to emerging new variants [6] and (iii) high-cost and need for specific cold chain suppliers as observed with recent mRNA vaccines against SARS-CoV-2. Although developing vaccine strategies against the respiratory diseases still remain an outstanding priority, it questions the availability of these vaccines in developing countries in a timely manner, where cost-effective antiviral therapies could be preferred.

### 2.1. Symptomatic Treatment

For most respiratory viruses, the guidelines for their treatment and management can differ widely according to the patient status or the country [21]. Most vRTIs medications are not antiviral agents but rather target the short-term relief of symptoms, such as nasal decongestants, antipyretics/analgesics, antitussives or expectorants. However, though rare, adverse effects could occur, especially in young children [6,22]. Therefore, using targeted therapeutic options against these respiratory viruses is highly required.

### 2.2. Antiviral Medication

In general practice, the treatment is done with nonspecific antiviral agents, e.g., cidofovir against adenovirus infections, ribavirin against influenza, and palivizumab for protection against RSV (Table 1). The use of adamantane drugs and neuraminidase inhibitors (oseltamivir, laninamivir, peramivir and zanamivir) has been also reported in the literature against influenza [6,23].

Although active research is ongoing to develop safe and efficient antiviral agents against respiratory viruses [28], their clinical effectiveness is still under investigation, and their use is limited to high-risk populations, such as immunocompromised patients [29]. These antiviral agents lack targeted therapeutic activity towards respiratory viruses. Their cost, as well as associated side effects, restrict their use. Moreover, the emergence of viral resistance remained a major limiting factor for the general population and emerged as a major public health problem [30,31]. Furthermore, using antibiotics is still widely practiced in certain parts of the world, which leads to their misuse [32]. Due to the lack of optimal medication and effective vaccines, the search for alternative natural therapies, such as sulfated marine polysaccharides, is indispensable.

## 3. Antiviral Activity of Sulfated Polysaccharides and Their Underlying Mechanisms of Action

**Figure 2:**
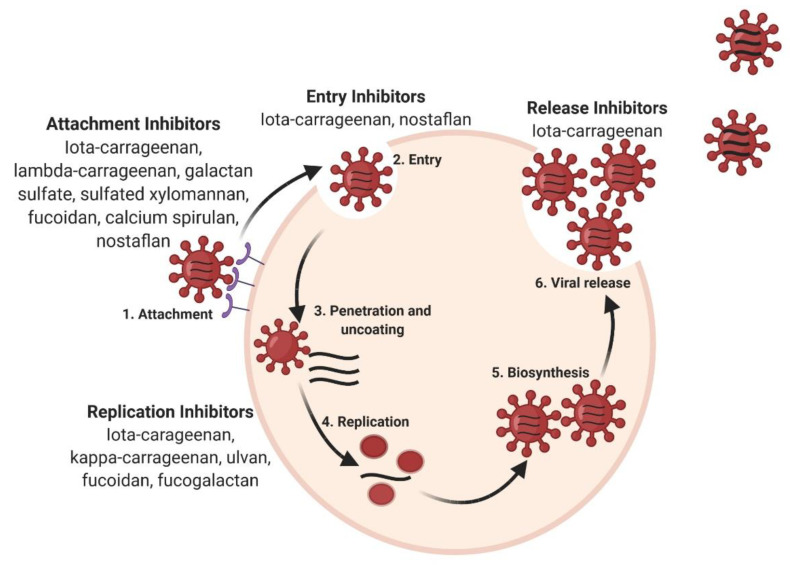
Mechanism of action of seaweed sulfated polysaccharides at various stages of the viral cycle.

Since the pneumonic plague, the research sector is exploring all the possible options to select promising antiviral drugs [33,34]. The search for appropriate antiviral agents is not only limited to synthetic molecules, but other natural alternatives are also in consideration because of some interesting properties, such as biocompatibility, low toxicity, biodegradability and renewable nature [35,36]. In this context, natural products using terrestrial sources (herbal medicine) [37] or marine origin [20,36] have gained huge interest. The SARS-CoV-2 pandemic has given rise to many investigations of natural products to treat the associated respiratory disease. Among these products, seaweed polysaccharides have been in the highlights recently [38]. In fact, seaweed polysaccharides, particularly sulfated polysaccharides (SPS), were already known to exhibit potent antiviral activity against a wide variety of enveloped viruses, such as herpes simplex virus or human immunodeficiency virus [39,40] but also against the respiratory viruses. Their mechanisms of action are quite diverse, and they could act at different steps of the viral cycle, from preventing viral attachment to intracellular antiviral activity employing different pathways [33,41] (Figure 2).

The formation of specific supramolecular complexes of SPS, with the virus or the target cell, is the basis of their antiviral activity. These complexes rely on non-covalent interactions (electrostatic mainly, but also hydrophobic and polar) and are commonly attributed to the structural characteristics and composition of the SPS. Their degree of sulfation, molecular weight and structural features (presence of O-acetyl groups, specific positioning of sulfate/acetyl) are the key factors in their antiviral activity [42] as highlighted with chemically SPS [43,44].

### 3.1. Inhibition of Infection by Electrostatic Interaction

Negative charges borne by the sulfated groups can initiate electrostatic interactions that are key for antiviral activity. The presence of the sulfate groups could inhibit the binding process of the virus to its target cell, preventing viral attachment to the cell receptor at the very early stage of infection by inhibiting syncytium formation. The virus entry can be prevented by masking the positive charges of the viral receptor [45]. In the case of enveloped viruses, negatively charged SPS could interfere in the association of the positively charged glycoprotein of the viral envelope and the cell surface receptor [27,32]. The irreversible encapsulation of the viral particles by sulfated polysaccharides was observed by scanning probe microscopy on the HIN5 influenza virus [46].

### 3.2. Inhibition of Infection by Allosteric Interaction

Allosteric interaction is described as the binding of a ligand to an allosteric site of a receptor, inducing a conformational change. This modification can either induce positive or negative cooperativity in the binding of the orthosteric agonist. In the case of the viral infection, the polysaccharide is assimilated to the ligand and the virus to the orthosteric agonist. Then, the allosteric interaction of an SPS with the cell receptor can impede virus binding and subsequent internalization. In addition, by allosteric interaction, SPS can bind to the viral capsid, preventing the uncoating of the virus inside the host cell [38].

### 3.3. Modulation of Intracellular Antiviral Activity

SPS have been shown to inhibit intracellular processes, such as viral replication. For example, as shown for HIV infection, SPS can interfere with replication enzymes, such as reverse transcriptase, or prevent the translation of proteins [47]. As seen particularly in influenza, SPS can stimulate cellular pathways that inhibit viral infection [41,45,48]. Indeed, SPS could specifically bind to the Toll-like receptors (TLRs) that are involved in the innate immune response to microbes [49]. This binding will induce the secretion of proinflammatory cytokines and provoke an antibody-mediated adaptive immune response, promoted by the activation of immune cells [50].

## 4. Antiviral Activity of Seaweed Sulfated Polysaccharides against Respiratory Viruses

Marine SPS are natural drugs that exhibit broad-spectrum antiviral activity and can be a promising solution to emerging viral infections, especially respiratory infections [51,52]. To analyze the antiviral activity of marine polysaccharides, and in particular seaweed SPS against the respiratory viruses, the experimental setup widely used is based on in vitro assays using specific cell lines. These assays use a viral source as the starting point and an appropriate model cell line for the viral infection. By monitoring the kinetics of infection in the presence of the drugs, the therapeutic and preventive potential of these polysaccharides is assessed. These assays also aim at determining the underlying mechanism of action of seaweed SPS using time of addition assays, where the polysaccharide is added before, during or after the viral infection. In certain cases, animal models are also used to assess the antiviral efficacy after the viral challenge. However, this in vivo approach is limited to a few respiratory viruses, such as the influenza virus [53] or adenovirus [54], as biosafety issues related to these animal models could limit the access to strong preclinical data.

Yet, data collected from literature could permit to broadly classify the antiviral activity of the marine polysaccharides obtained from seaweeds based on their capacity to inhibit the respiratory viral infection, either in vitro or in vivo, as illustrated in Table 2.

Thus, according to their mechanism of action during the viral infection cycle, either entry inhibition or viral replication inhibition, we could provide a tentative classification between polysaccharides from four marine algae.

Moreover, the following sections aim at deciphering the nature of seaweed SPS, classified according to specific respiratory viruses, against which they exhibit antiviral activity.

### 4.1. Coronaviruses

The viruses of the CoV family are single-stranded RNA-enveloped viruses. They are divided into 4 classes (alpha-CoV, beta-CoV, gamma-CoV and delta-CoV) and are responsible for about 20% of the common human colds [79]. Several studies reported the antiviral efficacy of seaweed polysaccharides against beta coronaviruses (human coronavirus hCoV OC43 and SARS-CoV-2).

Iota-carrageenan containing lozenges were reported to inhibit the viral activity of hCoV OC43 [70]. The antiviral activity of this red algae polysaccharide was explained by its polyanionic nature that inhibits the virus adsorption. In another instance, Kwon et al. 2020 showed that fucoidan fractions from brown algae, *Saccharina japonica,* can efficiently inhibit in vitro entry of SARS-CoV-2 in Vero cells due to multivalent interactions between the polysaccharide and viral particles [72].

Seaweed SPS from different sources were tested for their antiviral competency against SARS-CoV-2. Among those sulfated polysaccharides, fucoidan and iota-carrageenan from brown and red seaweed, respectively, showed antiviral potential against SARS-CoV-2 by preventing host cell entry in Vero E6 cells, thus inhibiting viral infection [69]. In a study comparing the antiviral activity of different SPS from seaweeds, iota-carrageenan was shown to possess maximum antiviral activity against SARS-CoV-2 [68]. These antiviral assays were conducted in vitro on ACE2-HEK293 cells using SARS-CoV-2 spike pseudotyped lentivirus and were shown to inhibit both the viral attachment as well as replication. Furthermore, Bansal et al. confirmed the antiviral activity of iota-carrageenan nasal spray in vitro using Vero E6 cells [67]. Lambda-carrageenan showed the potent inhibition of SARS-COV-2 in vitro in Vero cells, preventing the viral attachment to cell receptors [64].

### 4.2. Influenza Virus

Influenza viruses belong to the Orthomyxoviridae family and are classified into 4 types (A, B, C and D). Flu is an infectious respiratory disease caused in humans by influenza A or B viruses. Influenza A viruses are divided into subtypes based on the two proteins of the viral envelope: hemagglutinin (H) and neuraminidase (N). Hemagglutinin has 18 different subtypes (H1 to H18) and neuraminidase 11 (N1 to N11). To date, 131 subtypes (different combinations of H and N) have been detected in nature. H1N1 and H3N2 are the current subtypes of influenza A viruses that routinely circulate in humans. Every 10-50 years, a new influenza A virus strain leads to a pandemic [30]. Only a safe and broad-spectrum antiviral medication could stop influenza propagation without an effective and universal vaccine.

k-carrageenan from red algae has proven to prevent antiviral activity against the influenza A virus (H1N1) in MDCK cells. This seaweed SPS has been shown to interfere in the early replication steps of the virus [41,80]. Furthermore, hybrid carrageenan from red algae, *Eucheuma denticulatum*, has also shown antiviral activity against the influenza virus (H1N1) [65]. Iota-carrageenan was demonstrated to inhibit virus entry in the cells by direct interaction with virus particles. This electrostatic interaction can inhibit viral adsorption on cellular receptors, thus inhibiting syncytium formation. This study has been conducted on MDCK, Vero and primary human nasal epithelial cells (HNep). The antiviral efficacy of the nasal application of iota-carrageenan has also been shown in infected mouse models [53] and randomized trial data [56]. Lambda-carrageenan inhibited both influenza A and B viruses in vitro on MDCK cells along with infected mouse models. This molecule has shown its effect by preventing the viral attachment to cell receptors [64].

Polysaccharide fractions from *Gracilaria lemaneiformis* have also revealed the potential to inhibit viral adsorption and replication of human influenza virus H1-364 on MDCK cells [66]. Furthermore, immunomodulatory effects of polysaccharide fractions from *Sargassum qingdaoens* and *Grateloupia filicina* were highlighted in MDCK cells, as well as in mouse model infected by the avian influenza virus (AIV) (H9N2) [81]. In this study, the polysaccharides from microalgae were shown to inhibit the activity of AIV in vitro and to have stimulatory effects on the immune system after immunization of mice with an inactivated AIV. These immunomodulatory properties of SPS could also have interesting applications, such as vaccine adjuvants.

Galactan sulfate from *Aghardhiella tenera* and sulfated xylomannan from *Nothogenia fastigiata* presented broad antiviral activity against the influenza infection by preventing virus adsorption on host cells, as demonstrated on several strains of enveloped viruses, including influenza [59,60].

Among brown algae, fucoidan from *Laminaria japonica* has proven to encapsulate viral particles leading to its in vitro inactivation, thus preventing cell infection by AIV (H5N1) [46,73]. Fucoidan extract from *Kjellmaniella crassifolia* has been shown to prevent in vitro and in vivo replication of the influenza A virus (H1N1 and H3N2) through inhibition of the viral neuraminidase and cellular epidermal growth factor receptor (EGFR) pathway [48]. The broad antiviral effects of this extract have been shown to be superior to the anti-influenza A virus drug amantadine when tested on MDCK and human lung epithelial cells A549, as well as in infected mouse model through nasal application of fucoidan [48].

Moreover, fucogalactan from *Undaria pinnatifida* has shown to inhibit the in vivo replication of the influenza A viruses in mice infected models by decreasing viral replication and increasing humoral (neutralizing antibodies) and innate (natural killer and macrophages activities) immunity pathways [42,50]. Similarly, Akamatsu et al. showed the anti-influenza activity of fucose polysaccharide MC26 [82].

Polysaccharides from green algae, *Coccomyxa gloeobotrydiformis* and *Ulva lactuca* have also been shown to possess anti-influenza activity by inhibiting viral-cell fusion with several influenza A viruses (such as H1N1, H2N2, H3N2, H1N1 pandemic strains) and replication in avian and human influenza viruses, respectively, in MDCK cells [74,75].

### 4.3. Human Parainfluenza Virus

HPIV belongs to the Paramyxoviridae family and shares common features with the influenza virus, such as hemagglutinin and neuraminidase spikes. They are single-stranded RNA viruses divided into four types (1, 2, 3 and 4). All forms of HPIV provoke infection of either the upper or lower respiratory tract, with associated symptoms similar to the common cold (such as wheezing, coryza, rhonchi, otitis) [83]. Unlike the influenza virus that spreads mainly during winter, parainfluenza viruses are also widespread between spring and autumn. HPIV is an enveloped virus, and as many respiratory enveloped viruses, seaweed polysaccharides can inhibit viral binding to the cell and thus inhibit virus–cell fusion because of their polyanionic nature that masks the virus.

Witvrouw et al. 1994 showed that galactan sulfate from red algae, *Aghardhiella tenera,* can inhibit the binding of HPIV 3 to the host cell in vitro on Vero/HeLa cells [59].

Fucoidan from brown algae *Laminaria japonica* has also proven antiviral activity against HPIV 1 [54]. This polysaccharide has been studied in vitro on MDCK cells and in vivo, using an infected mouse model. A prolonged survival time of mice infected with HPIV 1 was shown after intravenous injection of low molecular weight fucoidan. Interestingly, the study by Sun et al. highlighted that, besides inhibition of virus–cell contact, the in vivo antiviral activity of fucoidan could also be attributed to the improvement of humoral and cellular immunity [54].

### 4.4. Respiratory Syncytial Virus

RSV is a single-stranded RNA virus from the family of Pneumoviridae. RSV is divided into two subtypes, A and B, which cause mild cold-like symptoms. RSV is one of the most common causes of bronchiolitis and pneumonia and can have serious consequences in infants or the elderly [12].

Galactan sulfate from *Aghardhiella tenera* and sulfated xylomannan from *Nothogenia fastigiata* is known to inhibit the RSV viral entry to the cell surface receptor [59,60]. In addition, the ethanolic extract of polysaccharides obtained from brown algae *Laminaria japonica* is known to inhibit viral replication in HEK293 cells [71].

### 4.5. Human Metapneumovirus

HMPV is a single-stranded RNA virus from the Pneumoviridae family. After RSV, HMPV is the second leading cause of lower respiratory tract infections in infants.

Iota-carrageenan is also known to inhibit the in vitro replication of HMPV in the rhesus monkey kidney epithelial cell line, LLC-MK2, by blocking the virus release from the cell surface, either by modifying the plasticity of the membrane or by anchoring the progeny virions to the cell [57].

Various extracts obtained from green algae, *Ulva fasciata*, have been shown to inhibit both the viral entry and replication of HMPV in vitro using Vero cells. This study highlighted the impact of different environmental and extraction conditions on the antiviral activity of HMPV [76].

### 4.6. Human Rhinovirus

HRV belongs to the picornavirus family. HRVs are non-enveloped single-stranded RNA viruses divided into three species (A, B and C). There are around 160 recognized serotypes of HRVs differing from their surface proteins. The existence of such important variability in serotypes makes developing a broad antiviral drug extremely difficult and challenging.

The sulfated seaweed polysaccharide iota-carrageenan from red algae inhibits HRV infection due to its electrostatic interaction with the cell surface receptor. Among the widely studied carrageenan derivatives, iota-carrageenan is the most important antiviral polysaccharide against various respiratory viruses. The in vitro effectiveness of this polysaccharide has been tested on HeLa and human primary nasal epithelial cells on various strains of human rhinovirus [53,84]. This polysaccharide is also known to suppress the viral replication of various non-enveloped viruses [45,70].

Iota-carrageenan is the only approved natural seaweed antiviral against HRV. The efficacy and safety of the nasal spray against HRV were tested in controlled clinical trials in patients with common cold [85,86]. The therapeutic effectiveness against common cold patients was also analyzed from two randomized, double-blind placebo-controlled trials [56,87]. The non-clinical safety evaluation of intra-nasal formulation of iota-carrageenan has been conducted on rabbits and rats. These studies were performed according to ISO 10993 “Biological evaluation of medical devices” and current European and OECD guidance for testing pharmaceuticals for human use [88].

### 4.7. Adenoviruses

Adenoviruses are members of the family Adenoviridae. They are non-enveloped double-stranded DNA viruses. More than 200 adenoviruses have been officially recognized and are mostly isolated from humans, even though they can infect other mammals, reptiles, birds, fish, and amphibians [89].

The sulfated seaweed polysaccharide fucoidan obtained from red algae, *Laminaria japonica*, has proven to show antiviral activity against adenovirus [53]. The antiviral activity of this polysaccharide has been studied in vitro on Hela cells and in vivo, using an infected mouse model. The antiviral activity of this polysaccharide is attributed to its polyanionic nature, which masks the virus or positive region of cell surface inhibiting the virus adsorption. IV injection of low molecular weight fucoidan showed a prolonged survival time of virus-infected mice [53]. Another polysaccharide extract from red algae, *Pterocladia capillacea*, has been shown to inhibit the viral replication of adenovirus 40 in vitro on human larynx carcinoma cell lines (Hep-2) [58].

## 5. Limits to be Overcome for the Use of Seaweed Polysaccharides against Viral Respiratory Infections

As the seaweed SPS possesses a broad antiviral spectrum, it could have an added benefit over other antiviral agents used against respiratory infections due to its safety and nontoxic nature. However, despite a considerable amount of research regarding their antiviral potential, mainly demonstrated through in vitro cell culture assays, the practical application of these seaweeds goes unrecognized. Indeed, to date, there has been limited clinical studies that highlight the effectiveness of seaweed SPS as treatment of infected individuals, restricted to iota-carrageenan [20,85,86,87,88] (Table 1), which is the only sulfated seaweed polysaccharide that has been approved by EMA for its use in common cold [20,84]. Thus, despite their promising potential, their use is mainly limited to nutraceuticals and cosmeceuticals [90,91]. Certain hurdles must be overcome to promote research into using these safe, natural polysaccharides in respiratory viral infections. The major limiting factors for their use as antiviral candidates in respiratory infections are discussed below.

### 5.1. Variability in Composition: Physicochemical Characterization

One of the main concerns for using sulfated polysaccharides is the heterogeneity of the final extract. Seaweed polysaccharides are complex heterogeneous molecule mixtures that can show variability in their composition, altering the physicochemical characteristics of the active compound. This variability can be attributed to the source of polysaccharide (e.g., species, time of harvest), environmental factors (light, nutrition, salinity, temperature), extraction and purification procedures. All these physicochemical factors can hugely impact the safety, efficacy, and pharmacological activity of these polysaccharides [92,93,94]. To promote their usefulness in clinical settings for a particular condition, guaranteed reproducibility in its physicochemical characteristics is the ultimate prerequisite. This pharmaceutical quality can be achieved by an efficient screening of active compounds in the seaweed extracts.

### 5.2. Complexity of Origin and Sustainability

The vast biodiversity of the ocean is difficult to analyze, and the huge biodiversity leads to accessibility issues for marine organisms. Hence, a subsequent major challenge faced for natural seaweed polysaccharides is ensuring their sustainable supply [95]. The slow growth rate, seasonality and low extraction yields are the major limiting factors in this regard. This problem can be overcome by using efficient screening technologies that are leading to faster identification of the active compound. To achieve this, efficient analytical tools like mass spectrometry, nuclear magnetic resonance can contribute to the rapid identification of the active component [96,97]. Identifying these active entities can allow the de novo structure determination even in very small concentrations in complex crude extracts [98]. Once the active polysaccharide is identified, the sustainability issue can be addressed by promoting the semi-synthesis of these active polysaccharides, such as selective sulfation [99,100]. However, one of the most important parameters is to confirm if this artificial synthesis can maintain the production of the desired active compound.

Identifying these lead compounds could avoid the problem of dereplication or “rediscovery of the known”, which can be cost-effective and time-consuming [95,101]. Furthermore, it can also ensure the reproducibility of active molecules with detailed physicochemical characteristics.

### 5.3. Need for an Improved Methodology for Antiviral Properties Assessment

Despite the greater influence and promising research towards natural marine products, there are only eight FDA/EMEA approved drugs in the pharmaceutical pipeline [102], with only one being approved for vRTIs. The major reason behind the discontinuation of compounds from clinical trials is the lack of high in vivo efficacy. As a matter of fact, as in vitro efficacy does not always predict in vivo efficacy, and improved preclinical methodology is required to access phase I/II short clinical trials, as exemplified by identifying new treatments against SARS-CoV-2. Furthermore, better crosstalk between basic science and industry through introducing well-designed research tools could favor a more comprehensive interpretation of data gained by academic researchers.

## 6. Conclusions

Besides other various biological activities, sulfated seaweed polysaccharides have proven their broad-spectrum potential against various respiratory illnesses. This review enlisted the potential of seaweed SPS as antiviral agents against various respiratory viruses. In the discussed examples, it can be seen that the antiviral efficacy of these seaweed SPS is well documented through in vitro assays, which have identified common mechanisms between the different seaweed polysaccharides. All these efforts can help provide practical application and deliver natural, safe options to combat respiratory viral infections. Thus, modulating the research criteria according to their purity, sustainability, and in vivo biological activity can increase the usefulness of these natural candidates. Their proven potential should be extended through preclinical research to combat respiratory infections/epidemics efficiently.

## Figures and Tables

**Table 1:** Some approved synthetic antiviral agents and natural seaweed polysaccharides against respiratory viruses.

* FDA/EMA Approved Antivirals				
Respiratory Virus	Synthetic Antivirals	Natural Seaweed Polysaccharides	Vaccine	Reference
Human rhinovirus	No	Iota-carrageenan	No	
Adenovirus	Cidofovir, ganciclovir, ribavirin	No	Yes (not accessible to all)	[24]
Human metapneumovirus	No	No	No	
Parainfluenza virus	No	No	No	
Influenza virus	Ribavirin, amantadine	No	Yes	[23]
	Rimantadine, zanamivir			
	Oseltamivir, peramivir			
	Laninamivir			
Respiratory syncytial virus	Ribavarin, palivizumab	No	No	[25,26]
SARS CoV-2	Remdesivir	No	Yes	[27]
HCoV-OC43	No	No	No	

* FDA: US Food and Drug Administration, EMA: European Medicines Agency.

**Table 2:** Antiviral activity of seaweed sulfated polysaccharides against respiratory viruses based on their mechanism of action (entry or replication inhibition).

Marine Algae	Polysaccharide	Respiratory Virus	Entry Inhibition	Replication Inhibition	Reference
Red Algae	Iota-carrageenan	HRV	×		[55,56]
	Iota-carrageenan	HMPV		×	[57]
	Iota-carrageenan	Adenovirus		×	[58]
	Galactan sulfate	HPIV	×		[59]
	Galactan sulfate	Influenza	×		[59]
	Sulfated xylomannan		×		[60]
	kappa-carrageenan			×	[45,61,62,63]
	Iota-carrageenan		×		[53,56]
	Lambda-carrageenan		×		[64]
	hybrid carrageenan (ı/κ/ν-carrageenan)		Not mentioned		[65]
	Polysaccharide fractions		×	×	[66]
	Galactan sulfate	RSV	×		[39,59]
	Sulfated xylomannan		×		[60]
	Iota-carrageenan	SARS CoV-2		×	[67,68]
	Iota-carrageenan		×		[68,69]
	Lambda-carrageenan		×		[64]
	Iota-carrageenan	HCoV-OC43	×		[56,70]
Brown Algae	Fucoidan	Adenovirus	×		[54]
	Fucoidan	HPIV	×		[54]
	Extract	RSV		×	[71]
	Fucoidan	SARS CoV-2	×		[69,72]
	Fucoidan	Influenza	×		[41,54,73]
				×	[48]
	Fucogalactan			×	[42,50]
Green Algae	Acidic polysaccharide	Influenza	×		[74]
	Ulvan polysaccharide			×	[75]
	Extracts	HMPV	×	×	[76]
Blue-GreenAlgae	Calcium spirulan	Influenza	×		[77]
	Nostaflan		×		[78]
